# Neutrophil extracellular trap clearance by synovial macrophages in gout

**DOI:** 10.1186/s13075-021-02472-4

**Published:** 2021-03-19

**Authors:** Ji Hye Jeong, Su Jin Choi, Soo Min Ahn, Ji Seon Oh, Yong-Gil Kim, Chang-Keun Lee, Bin Yoo, Seokchan Hong

**Affiliations:** 1 Division of Rheumatology, Department of Internal Medicine, University of Ulsan College of Medicine, Asan Medical Center, 88, Olympic-ro 43-gil, Songpa-gu, Seoul, 05505 South Korea; 2 Asan Institute for Life Sciences, Asan Medical Center, Seoul, South Korea; 3 Clinical Research Center, Asan Medical Center, Seoul, South Korea

**Keywords:** Monosodium urate crystals, Gout, Neutrophil extracellular traps, Efferocytosis

## Abstract

**Background:**

Monosodium urate (MSU) crystals, i.e., the central etiological factors in gouty arthritis, induce the formation of neutrophil extracellular traps (NETs). We investigated whether synovial macrophages could clear NETs as a self-resolution mechanism in acute gouty arthritis.

**Methods:**

Synovial fluid mononuclear cells (SFMCs) were incubated with NETs induced by MSU crystals. NET engulfment was determined based on neutrophil elastase (NE), myeloperoxidase (MPO), and SYTOX Green signals within synovial fluid CD14^+^ cells. In addition, the correlations between CD14^+^ cells, MPO-dsDNA complexes, and expression of pro- and anti-inflammatory cytokines were analyzed in the synovial fluid CD14^+^ macrophages of patients with gouty arthritis.

**Results:**

Synovial fluid CD14^+^ macrophages significantly engulfed the MSU crystal-induced NETs, as evidenced by the alteration in SYTOX Green intensity or the presence of NE and MPO in the cytoplasm of CD14^+^ cells. The proportion of CD14^+^ macrophages was significantly and inversely correlated with levels of MPO-dsDNA complex in the synovial fluid of gout patients. Synovial fluid CD14^+^ macrophages cultured with NETs did not show a significant induction in pro- and anti-inflammatory cytokines.

**Conclusion:**

Synovial fluid macrophages may play an important role in the resolution of MSU crystal-induced gouty inflammation by clearing NETs without causing any significant immunological response.

**Supplementary Information:**

The online version contains supplementary material available at 10.1186/s13075-021-02472-4.

## Background

Gout is an acute, episodic inflammatory disease characterized by sudden and painful arthritis caused by the deposition of monosodium urate (MSU) crystals [1, 2]. Immunological responses to MSU crystals are responsible for acute severe joint inflammation in gouty arthritis [3–6]. Among the various types of immune cells, neutrophils, i.e., the first line of defense in the innate immune system, have been recognized as one of the major players in the generation of acute dramatic inflammation because of their rapid accumulation and activation in the affected areas. Neutrophils undergo a process of neutrophil extracellular traps (NETs), a strategy to combat extracellular pathogens in response to stimuli, including MSU crystals [7]. NETs are composed of extracellular structures such as DNA, histones, and neutrophil granules (containing neutrophil elastase (NE) and myeloperoxidase (MPO)). Whereas NETs induced by MSU crystals can assemble pro-inflammatory responses, aggregated NETs can inhibit inflammatory responses through the degradation of IL-1β and IL-6 [8, 9]. Thus, neutrophils and/or NETs play an important role in the initiation and resolution of acute inflammatory diseases such as gouty arthritis [10].

Monocytes/macrophages are also known to play a role in the progression and resolution of gouty inflammation. Tissue-resident macrophages are responsible for the production of pro-inflammatory cytokines IL-1β, TNF-α, and IL-6 during the early phase of inflammation; later, the circulating monocytes infiltrate and differentiate into pro-inflammatory macrophages in MSU-peritonitis mouse models [11, 12]. In contrast, monocytes can acquire anti-inflammatory properties, for example by releasing transforming growth factor-β1 (TGF-β1), in response to MSU-crystals [13]. In addition, our recent study showed that monocytes/macrophages from the synovial fluid of gout patients exhibit anti-inflammatory as well as pro-inflammatory properties [14]. However, little is known about the interaction between immune effectors during the resolution process of gouty arthritis. In this respect, it remains largely unknown as to how immune cells recognize and respond to NETs generated in response to MSU crystals. Given that recent studies showed that macrophages are involved in clearing NETs by engulfing and degrading them [15–17], we here investigated whether monocytes/macrophages in the joints are involved in NET clearance in gouty arthritis.

## Patients and methods

### Study subjects

Synovial fluids were obtained from patients who were diagnosed with gout (*n* = 25) and osteoarthritis (OA) (*n* = 10) at a tertiary referral hospital in Seoul, South Korea. The diagnosis of gout was confirmed using polarizing microscopy to ascertain the presence of needle-shaped MSU crystals in the joint fluid. We included patients with OA who were diagnosed based on the American College of Rheumatology classification criteria for OA knee [18]. The clinical and laboratory data of the study patients are summarized in Table 1. Written informed consent was obtained from all patients and the study protocol (2016-0036) was approved by the Asan Medical Center Institutional Review Board.

**Table 1:** Clinical characteristics of patients with gout or osteoarthritis

	Gout (*n* = 25)	OA (*n* = 10)	*P*
**Age** (years, median [IQR])	63 (50–78)	62 (56–66)	0.694
**Male** (%)	25 (100%)	4 (40.0%)	< 0.001
**CRP** (mg/dL, median [IQR])	12.08 (4.44–17.75)	0.22 (0.10–1.00)	< 0.001
**Uric acid** (mg/dL, median [IQR])	6.0 (5.1–9.3)	5.0 (4.1–6.7)	0.137
**Involved joint**			
Knee	24 (96.0%)	10 (100%)	1.000
Ankle	8 (32.0%)	1 (10.0%)	0.234
Foot	5 (20.0%)	0 (0%)	0.291
**Treatment**			
IA steroid	19 (76.0%)	3 (30.0%)	0.019
Systemic steroid	11 (44.0%)	1 (10.0%)	0.112
NSAIDs	9 (36.0%)	7 (70.0%)	0.131
**Urate-lowering therapy**			
Allopurinol	2 (8.0%)	0 (0%)	0.579
Febuxostat	6 (24.0%)	0 (0%)	0.151
**Joint fluid analysis**			
WBCs (/μL, median [IQR])	33,500 (20750–53,955)	150 (74–554)	< 0.001
Neutrophil (/μL, median [IQR])	28,800 (18184–49,206)	2 (0–19)	< 0.001

*CRP* C-reactive protein,* IA* intraarticular, *WBCs* white blood cells

### Reagents and antibodies

Uric acid, lipopolysaccharide (LPS) (O111:B4), and NLRP3 inflammasome inhibitor (CP-456773) were purchased from Sigma (St Louis, MO). Quant-iT PicoGreen dsDNA Assay Kit was purchased from Thermo Fisher Scientific (Waltham, MA). RPMI-1640, penicillin-streptomycin, and fetal bovine serum were obtained from WelGene (Daejeon, South Korea). For multi-color flow cytometry, the following antibodies were used: Fixable Viability Dye eFluor® 506, anti-CD3 (UCHT-1), anti-CD19 (HIB19), anti-CD56 (B159), and anti-IL-1RA (CRM17) (All from eBioscience, San Diego, CA, USA); anti-CD14 (MφP9), anti-IL-1β (AS10), and anti-TNF-α (Mab11) (All from BD Bioscience, San Jose, CA); anti-IL-10 (JES3-9D7) and anti-IL-8 (6217) (Both from Biolegend, San Diego, CA); anti-TGF–β1 (27232) (R&D Systems, Minneapolis, MN). For immunofluorescence, SYTOX Green and anti-MPO (4A4) were obtained from Thermo Fisher Scientific (Waltham, MA). Anti-NE (NP57) and anti-citrullinated histone H3 (rabbit polyclonal) were obtained from Santa Cruz Biotechnology Inc. (Dallas, Texas) and Abcam (Cambridge, MA), respectively.

### Synovial fluid and synovial fluid mononuclear cell isolation

Aspirated synovial fluid samples were immediately transferred to the laboratory and synovial fluid mononuclear cells (SFMCs) were isolated using Ficoll-Paque™ PLUS gradient centrifugation (GE Healthcare, Piscataway, NJ). Supernatants were stored at − 80 °C until the measurement of MPO-dsDNA complexes.

### Neutrophil isolation and NET induction

After centrifugation of the peripheral blood using the Ficoll-Paque™ PLUS gradient, neutrophils were purified from erythrocyte-rich pellets using gradient sedimentation in a 3% dextran solution. Residual erythrocytes were then removed by hypotonic lysis. The purity of the isolated neutrophils based on the expression of CD66b and CD16 as assessed by flow cytometry was > 90%. Isolated neutrophils were cultured in Phenol-red–free RPMI-1640 medium supplemented with 2 mM l-glutamine, 100 U/ml penicillin, and 100 mg/ml streptomycin.

Freshly isolated neutrophils were incubated with MSU crystals (500 μg/ml) for 4 h. For engulfment ratio assessment and live cell imaging, neutrophils were incubated with MSU crystals in media containing SYTOX Green (SYTOX Green-labeled NETs). After incubation for 4 h at 37 °C, media containing MSU crystals were removed, and NETs were harvested by gently pipetting in fresh media. To retrieve NETs, centrifugation was performed at 450 *g* for 5 min followed by collection of the supernatants.

### Engulfment assay

SFMCs were seeded in RPMI-1640 medium with 5% FBS in a 96-well Imaging Plate (BD Falcon™) and incubated with SYTOX Green-labeled NETs for 6 h at 37 °C. This contraption was then treated with DNaseI (20 μg/ml, Worthington, Columbus, OH) for 10 min to digest the unengulfed NET complexes. The plate was washed three times with PBS, and fluorescence was measured using a Victor 3 microplate reader (Perkin Elmer, Waltham, MA, USA). The engulfment ratio of the NETs was determined as the ratio of relative fluorescence units of SFMCs incubated with SYTOX Green-labeled NETs to those of SFMCs alone.

### Immunofluorescence confocal microscopy

Isolated healthy neutrophils were seeded on poly-_D_-lysine coated cover slips followed by 1 h incubation for attachment. The neutrophils were incubated with MSU crystals for 4 h in the presence of SYTOX Green and then were fixed with 2% paraformaldehyde. After blocking with 3% bovine serum albumin (BSA), cells were probed overnight with anti-NE (1:200, Santa Cruz) [19], anti-MPO (1:200, Thermo fisher) [20], and anti-citrullinated histone H3 (1:200, Abcam) [21] at 4 °C. After staining with secondary antibodies, the cover slips were mounted using ProLong Glass Antifade Mountant (Invitrogen).

For imaging NET engulfment, SFMCs were incubated with purified NETs (not labeled with SYTOX Green) with or without MSU crystals for 6 h. To remove extracellular DNA, the cells were treated with DNaseI (20 μg/ml) for 10 min. After fixation with 2% paraformaldehyde and blocking with 3% BSA, cells were probed with anti-CD14 (HCD14) (Biolegend), followed by permeabilization and staining for NET components (NE and MPO). The stained cells were visualized using a ZEISS LSM780 confocal microscope (Carl Zeiss, Oberkochen, Germany).

For live-cell imaging, SFMCs were cultured in RPMI-1640 medium supplemented with 5% FBS in the presence of SYTOX Green-labeled NETs for 6 h and treated with DNaseI for 10 min, followed by attachment on a 35-mm confocal dish (NEST, Wuxi, Jiangsu, China). SFMCs were probed with CD14 antibody-conjugated Alexa® 594 and then visualized with a ZEISS LSM780 confocal microscope (Carl Zeiss) at 37 °C, 5% CO_2_. Images were analyzed using ZEN software (Carl Zeiss).

### MPO-dsDNA complex measurement

The MPO-dsDNA complex was measured as previously described with modifications. Briefly, anti-MPO antibody (4A4), as a capture antibody, was coated at 2.5 μg/ml in 96-well micro-plates. After washing, the plate was blocked with 1% BSA prepared in PBS. Synovial fluids were diluted 1:1 in PBS. To remove unbound DNA, the diluted synovial fluids were treated with DNase I followed by EDTA treatment [22]. Then, synovial fluids were loaded into the wells and incubated at 4 °C overnight. After washing, the Picogreen reagent was added and fluorescence was measured using a Victor 3 microplate reader (PerkinElmer) [23].

### Flow cytometric analysis

SFMCs were cultured at a density of 1 × 10^6^ cells/ml in a complete RPMI-1640 (Welgene) medium containing Golgi stop (BD Bioscience). Cells were challenged with various stimuli, such as NETs, LPS (1 μg/ml), or MSU crystals (200 μg/ml) for 6 h at 37 °C. To exclude dead cells, SFMCs were incubated with Fixable Viability Dye eFluor® 506 (eBioscience) at 4 °C for 30 min. After incubation with FcR blocking reagents (Miltenyi Biotec), primary antibodies were used to detect surface markers. Synovial macrophages were identified by expression of CD14 in the absence of CD1c after exclusion of CD3^+^CD15^+^CD56^+^ cells (Supplementary Figure 1). For evaluating the expression of intracellular cytokines by staining, cells were fixed and permeabilized according to the manufacturer’s protocol (eBioscience). Data were acquired on a BD FACSCanto™ II flow cytometer (BD Biosciences) and were analyzed using FlowJo software (Tree Star, Ashland, OR).

### Isolation of synovial fluid macrophages

After the removal of cell debris and dead cells from SFMCs using a dead cell exclusion kit (Miltenyi Biotec), synovial fluid macrophages were isolated with the monocyte isolation kit II (Miltenyi Biotec) according to the manufacturer’s protocol. The purity of the isolated synovial fluid macrophages was confirmed by flow cytometry staining with anti-CD14 antibody. The isolated cells were seeded in 96-well plates at a density of 2 × 10^6^ cells/ml and then stimulated for 6 h with NETs, MSU crystals, or LPS. After washout, cells were cultured for an additional 24 h, and then, the supernatants and cells were collected for ELISA and real-time PCR, respectively.

### Quantitative real-time PCR and ELISA

Total RNA was isolated with TRIzol (Invitrogen), and cDNA was synthesized using the SuperScript IV Reverse Transcriptase (Invitrogen). Quantitative real-time PCR was performed with TOPreal™ qPCR 2 × PreMIX SYBR Green (Enzynomics, Korea). Data are presented as relative mRNA levels normalized to the expression values of the reference gene (GAPDH). All reactions were run in triplicates. The concentrations of secreted cytokines in the collected supernatants were measured using the DuoSet ELISA kit (R&D Systems).

### Statistical analysis

Statistical analyses were performed using Prism 7 (GraphPad Software, San Diego, CA). The engulfment ratios of the NETs or levels of fluorescence intensity of SYTOX Green between groups were compared using a Wilcoxon test. Correlations were analyzed using Spearman’s rank correlation coefficients. Cytokine expression in CD14^+^ cells among groups was analyzed using an unpaired, nonparametric *t* test. Significance was defined as *P* <  0.05 (*), *P* <  0.01 (**), *P* <  0.001 (***), and *P* <  0.0001 (****).

## Results

### CD14^+^ macrophages in the synovial fluids of gout patients engulf MSU crystal-induced NETs

**Fig. 1:**
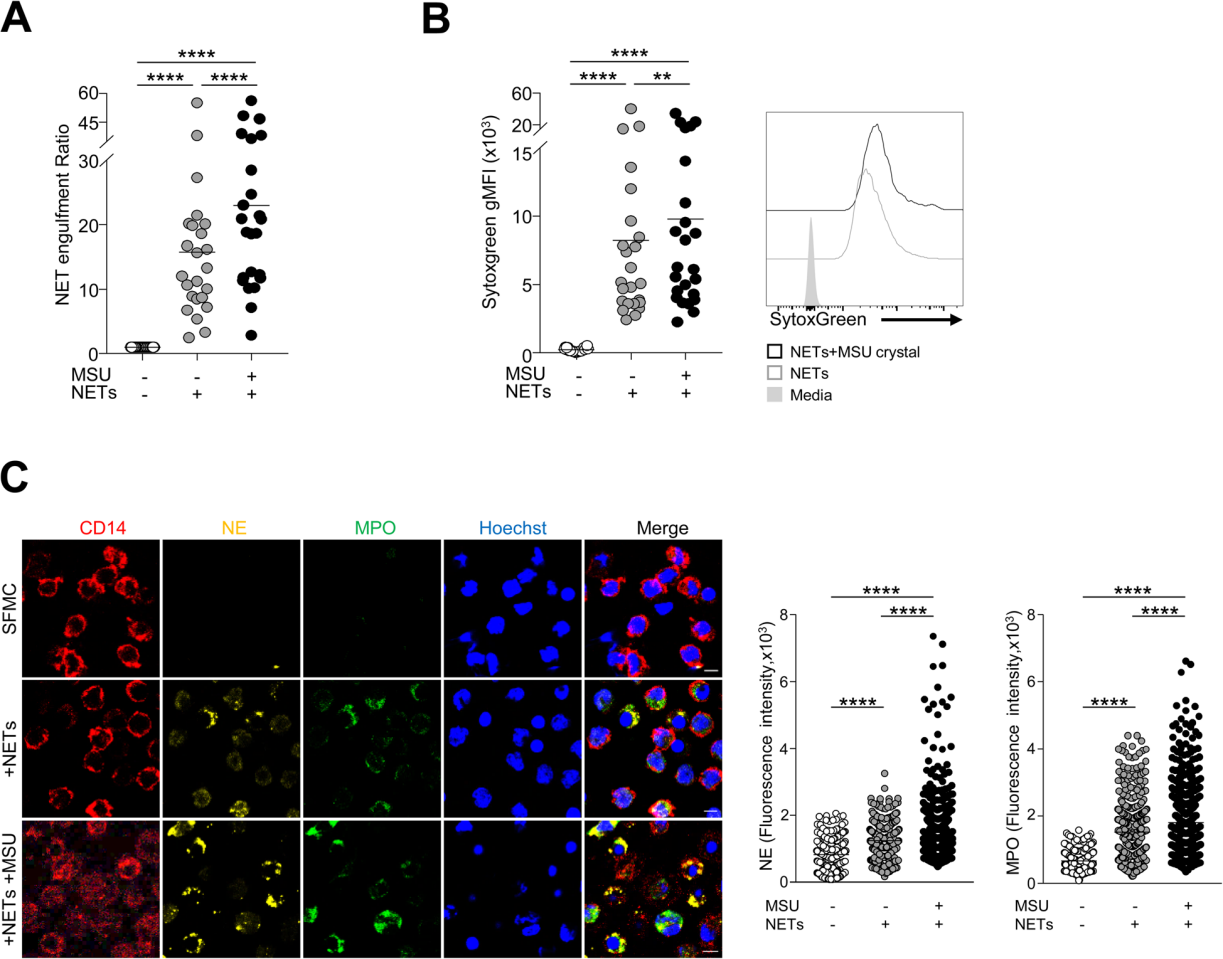
Engulfment of MSU crystal-induced NETs by synovial fluid macrophages. **a** Fluorescence units were measured after incubation of SFMCs with or without SYTOX Green-labeled NETs. The NET engulfment ratio was determined as the ratio of fluorescence units of SFMCs with NETs to that of SFMCs alone. When indicated, the engulfment ratio was calculated after exposure to MSU crystals during incubation. **b** Flow cytometric analysis of intracellular SYTOX Green in CD14^+^ macrophages (CD14^+^CD1c^−^CD3^−^CD19^−^CD56^−^ viable cells) from the SFMCs of gout patients. Statistical analysis (left) and representative plots (right) are shown. **c** NET uptake by synovial macrophages visualized based on the intracellular expression of neutrophil elastase (NE) (yellow) and myeloperoxidase (MPO) (green). Synovial macrophages were identified by staining for Hoechst 33342 (blue) and CD14 (red). Scale bar represents 5 μm. A quantitative comparison of NET formation measured by NE or MPO between the indicated conditions (right). gMFI, geometric mean fluorescence intensity. **P value < 0.01, ***P value < 0.001, ****P value < 0.0001

First, to examine whether neutrophils formed NETs in response to MSU crystals, we isolated peripheral neutrophils from healthy controls and incubated them with MSU crystals. Using confocal microscopy, we identified NETs based on NE, MPO, and citrullinated histone H3 expression in the extracellular space; these exhibited web-like structures (Supplementary Figure 2a). In addition, NE and MPO expression co-localized with SYTOX Green, a cell-impermeable nucleic acid staining dye, after stimulation with MSU crystals (Supplementary Figure 2b). Next, to address whether SFMCs from gout patients can uptake MSU crystal-induced NETs, we examined the fluorescence intensity after incubation with SYTOX Green-labeled NETs (Fig. 1a). NETs were significantly engulfed by the SFMCs. Interestingly, engulfment was further significantly increased in the presence of MSU crystals. In addition, we examined the intracellular levels of SYTOX Green to evaluate NET uptake by synovial fluid macrophages from gout patients. CD14^+^ macrophages in the synovial fluid of patients with gout displayed significant uptake of NETs, as evidenced by the significant levels of intracellular SYTOX Green in CD14^+^CD1c^−^CD3^−^CD15^−^CD56^−^ cells in flow cytometry. The ability of synovial fluid CD14^+^ macrophages to uptake NETs was significantly higher when exposed to MSU crystals during the incubation step (Fig. 1b). Furthermore, engulfment of NET components by the synovial macrophages was confirmed by the presence of NE and MPO in the cytoplasm of synovial fluid CD14^+^ cells when the images were obtained using confocal microscopy. Again, the presence of MSU crystals resulted in a more prominent uptake of NE and MPO by CD14^+^ cells (Fig. 1c). Considering that MSU crystals are well-known NRLP3 inflammasome activators, we examined the effect of the NLRP3 inflammasome pathway on NET uptake. We found that the NET engulfment ratio was not significantly affected by NLRP3 blockade, CP-456773 (Supplementary Figure 3). Collectively, these findings indicate that CD14^+^ macrophages in the SFMCs of patients with gout have a capacity to engulf NETs, particularly in the presence of MSU crystals.

### Synovial fluid macrophages from gout patients are inversely correlated with NET complex

**Fig. 2:**
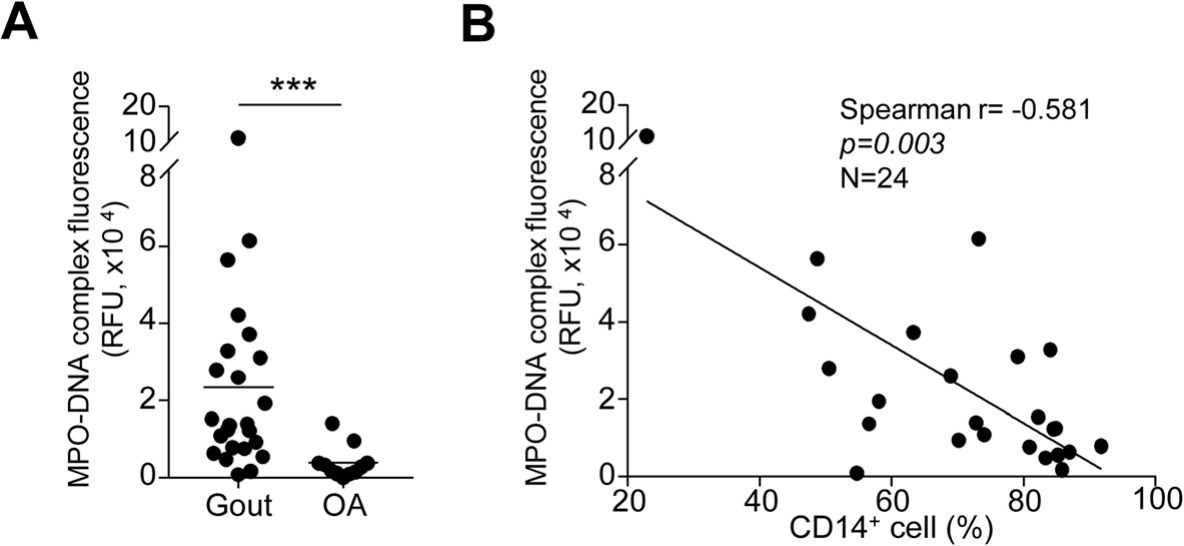
The correlation between synovial macrophages and NET clearance in gout patients. **a** The levels of NETs were measured based on fluorescence of the MPO-dsDNA complex in the synovial fluid of gout (*n* = 24) and OA (*n* = 10) patients. **b** Correlations between the proportion of CD14^+^ macrophages and levels of MPO-dsDNA complex. Statistical analysis was performed using unpaired Student’s *t* test (**a**) and Spearman’s rank correlation analysis (**b**). ***P value < 0.001

Next, we examined the correlation between NET complexes and synovial macrophages to determine whether CD14^+^ macrophages in gouty joints are associated with NET clearance. When we measured the levels of the MPO-dsDNA complex, which indicates the release of NET, in the synovial fluid of patients with acute gout, the levels were significantly higher in gout patients than those in OA patients (Fig. 2a). Further, we found that the levels of MPO-dsDNA complex were significantly negatively correlated with the proportion of CD14^+^ cells in SFMCs (Fig. 2b). These results suggest that CD14^+^ macrophages in synovial fluid of gout patients are significantly associated with NET clearance during gout attacks.

### Engulfed NETs are maintained within synovial fluid macrophages

**Fig. 3:**
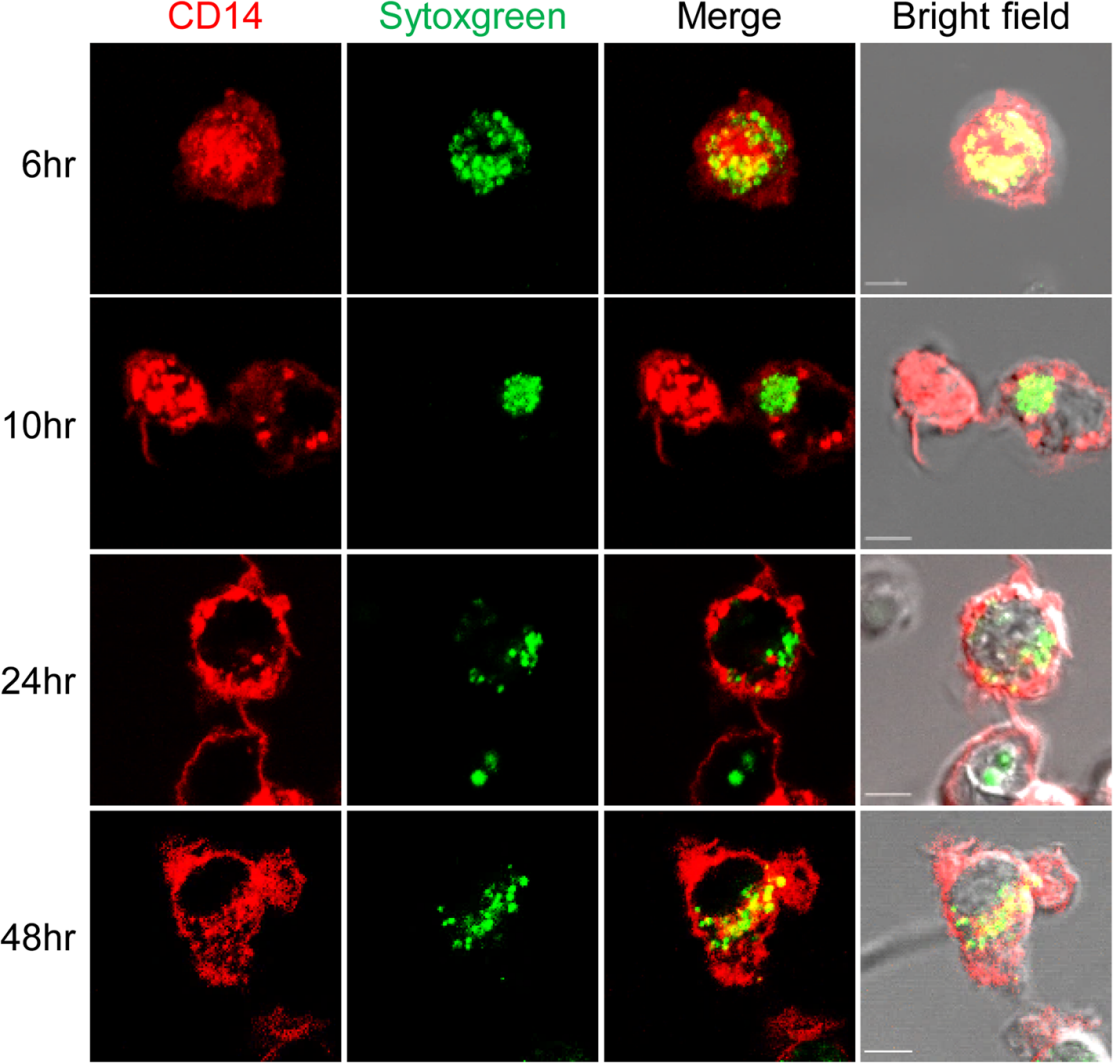
Maintenance of engulfed NETs within CD14^+^ cells from the synovial fluid of gout patients. SFMCs were incubated with SYTOX Green-labeled NETs and then treated with DNase I (20 μg/ml). At the indicated time points, the SFMCs were observed for the SYTOX Green signal within CD14 by confocal microscopy. Representative images are presented (*n* = 4). Scale bar represents 5 μm

Next, we investigated whether engulfed NET complexes were degraded by synovial fluid macrophages. The degradation of internalized NETs was determined by imaging SYTOX Green intensity after incubation with SFMCs and SYTOX Green-labeled NETs. We found that the fluorescence intensity of SYTOX Green within CD14^+^ cells was maintained for up to 48 h in culture. This showed that engulfed NETs were not immediately degraded within synovial macrophages (Fig. 3).

### Synovial fluid macrophages are maintained in an immunologically silent state after interaction with NETs

**Fig. 4:**
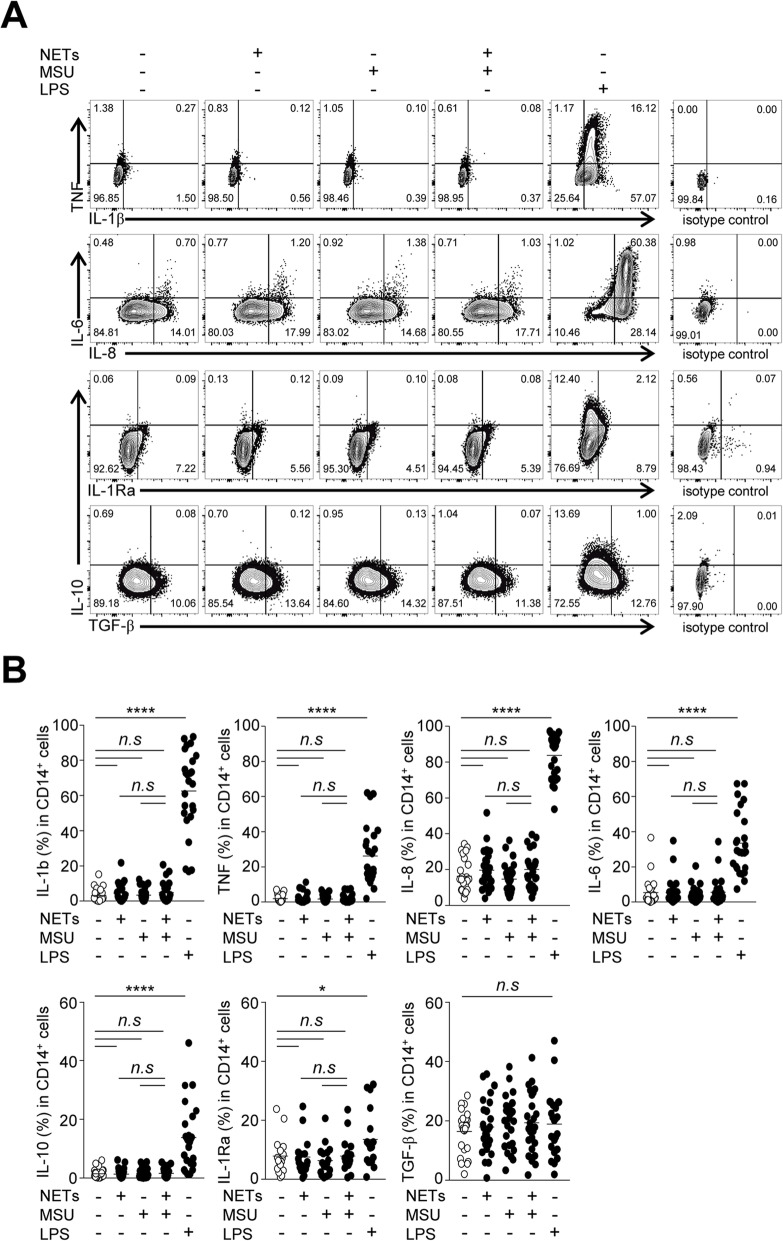
Cytokine expression in synovial fluid CD14^+^ macrophages in response to MSU crystal-induced NETs. SFMCs were analyzed for cytokine expression by flow cytometry after stimulation with or without NETs, MSU crystals (200 μg/ml), or LPS (1 μg/ml). Intracellular cytokine expression in CD14^+^ macrophages (CD14^+^CD1c^−^CD3^−^CD19^−^CD56^−^ viable cells) was determined, and **a** representative flow cytometry plots are presented. **b** The result of statistical comparison between the indicated groups using unpaired Student’s *t* test are presented. The analysis was performed in patients with gout (*n* = 25 except for IL-1Ra where *n* = 17). *P value < 0.05, ****P value < 0.0001

**Fig. 5:**
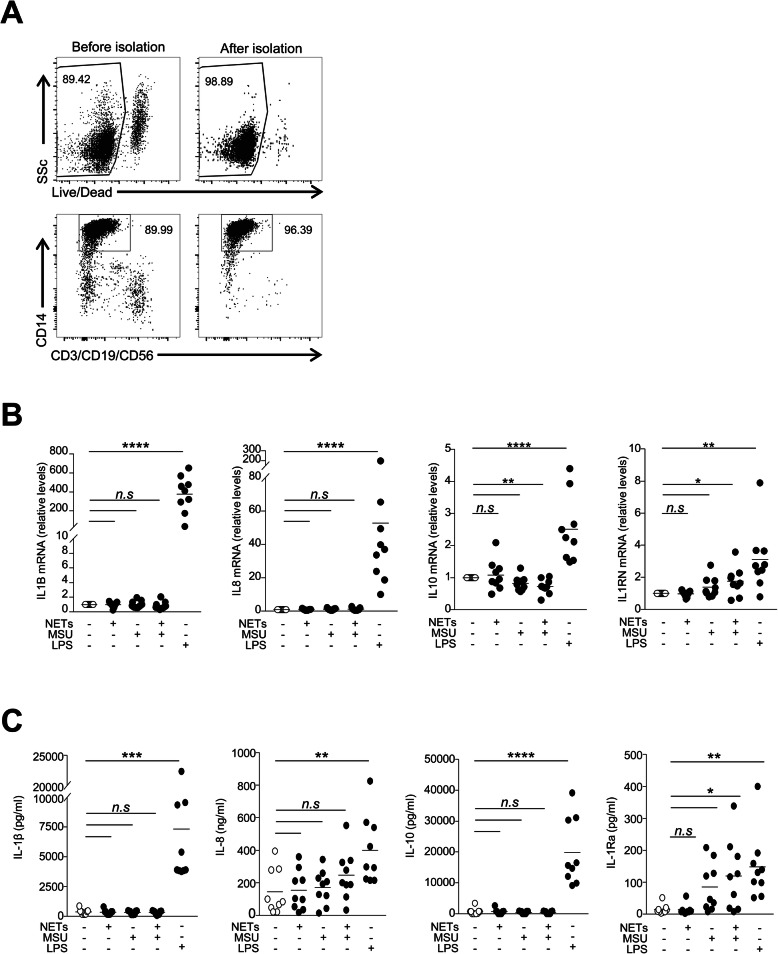
Cytokine expression in the isolated synovial fluid CD14^+^ macrophages. **a** Representative plots showing the purity of synovial fluid CD14^+^ macrophages isolated from patients with gout. **b** mRNA expression of cytokines measured by real-time quantitative PCR in isolated synovial fluid macrophages after stimulation. **c** Concentrations of secreted cytokines in the supernatants of isolated macrophages in response to NETs, MSU crystals, or LPS (*n* = 9). *P value < 0.05, ****P value < 0.0001

Given the persistence of the NET structure within macrophages, we next examined the immune responses of CD14^+^ cells during interaction with NETs. After stimulation with LPS, the levels of pro- and anti-inflammatory cytokines (except TGF-β1) were significantly increased in synovial fluid CD14^+^ macrophages of patients with gout. However, the expression of pro-inflammatory (IL-1β, TNF-α, IL-8, and IL-6) and anti-inflammatory cytokines (IL-10, IL-1Rα, and TGF-β1) was not significantly altered after stimulation with NETs and/or MSU crystals (Fig. 4a, b). Among the pro-inflammatory cytokines, intracellular IL-8 levels were slightly increased after stimulation with NETs, but this was not significant. Next, to address whether protease contained in neutrophils can degrade cytokines within macrophages, we incubated SFMCs in the presence of PMSF, a serine protease inhibitor, during co-culture with NETs and/or MSU crystals. However, the expression levels of pro-inflammatory and anti-inflammatory cytokines were not significantly altered according to the addition of PMSF (Supplementary Figure 4). Furthermore, when we examined the levels of transcripts and secreted proteins in synovial fluid macrophages isolated from patients with gout (Fig. 5a), we did not observe a significant induction of cytokines upon stimulation with NETs and/or MSU crystals except IL-1Rα levels (Fig. 5b, c). Taken together, these results indicate that NET uptake by the synovial fluid macrophages was not associated with significant pro- or anti-inflammatory responses.

## Discussion

In this study, we demonstrated that MSU crystal-induced NETs were engulfed by CD14^+^ macrophages, in particular in the presence of MSU crystals. A significant negative correlation was found between the proportion of macrophages and the amount of NET complexes in the synovial fluid of patients with gouty arthritis. In addition, the NETs within synovial fluid macrophages could be maintained without significant degradation and did not contribute to the significant pro- and anti-inflammatory immune responses.

It has been suggested that excess NETs play an important role in the pathogenesis of various rheumatic disorders including rheumatoid arthritis, systemic lupus erythematosus, and gouty arthritis [24–26]. As previously reported [27, 28], we found that MSU crystals can induce NET formation, as detected by immunofluorescence confocal microscopy (Supplementary Figure 2) and the detection of MPO-dsDNA in the synovial fluid of patients with gout (Fig. 2a). Recent studies have shown that NETs are cleared by macrophages such as human monocyte-derived macrophages [15, 16, 29]. In our present study, engulfment of NETs by synovial fluid macrophages was demonstrated by measuring the fluorescence of SYTOX Green-labeled NETs after culture with SFMCs. In addition, we showed that CD14^+^ macrophages from the synovial fluid of patients with gout can engulf MSU crystal-induced NETs, as evidenced by NE and MPO or SYTOX Green uptake in confocal microscopy or flow cytometry, respectively (Fig. 1b, c). Furthermore, the proportion of CD14^+^ cells was inversely correlated with the levels of the MPO-dsDNA complex in the synovial fluid of gout patients (Fig. 2b). Collectively, these results indicated that CD14^+^ macrophages in the synovial fluid of patients with gout play an important role in the removal of MSU crystal-induced NETs during gout attacks. Engulfed NETs can be maintained without degradation in the macrophages, but the evidence on when and how these structures are eventually processed within macrophages was not provided in our present study. Therefore, the long-term fate and effect of engulfed NETs in the pathophysiology of gout should be examined in a further study.

Defective efferocytosis of NETs has been suggested as one of the pathogenic mechanisms in non-infectious diseases [16, 29]. A previous study showed that impaired clearance of NET complexes may lead to systemic inflammation and tissue damage in an alcoholic liver disease model [29]. However, because it has not been examined in conditions of a specific blockade of NET clearance, the functional significance of the defective efferocytosis of NETs in disease pathogenesis is still unclear. In our study, we observed no significant pro- or anti-inflammatory responses from CD14^+^ macrophages in the synovial fluid of gout patients after interaction with the NET complexes. This is consistent with previous studies showing that macrophage uptake of NETs was not associated with the production of pro-inflammatory cytokines such as IL-β and TNF-α [15]. Thus, it is likely that the process of NET uptake by synovial fluid macrophages can be one of the mechanisms regulating the overwhelming immune response to NETs in gout. Further studies addressing the functional consequences of defective efferocytosis of NETs in the resolution phase of acute gout are needed.

Interestingly, the engulfment of NETs by macrophages was significantly higher in the presence of MSU crystals compared to that in the absence of MSU crystals (Fig. 1). These results suggest that the NET engulfment capacity of synovial fluid macrophages can be enhanced by MSU crystals. MSU crystals are well-known NLRP3 inflammasome activators [30], and it has recently been reported that the NLRP3 inflammasome and its effector, caspase-1, can regulate the phagosome function through the modulation of pH [31]. However, our results have shown that engulfment activity was not affected by the inhibition of the NLRP3 pathway (Supplementary Figure 3). Recent studies have provided possible mechanisms as to how NETs can be recognized and cleared by the immune system. Lazzaretto et al. [17] demonstrated that LL-37 enhanced the uptake of PMA-induced NETs, and degradation of NETs within macrophages was dependent on TREX1 (DNaseIII), an endoplasmic reticulum nuclease. It must also be considered that functional and compositional characteristics are different between NETs induced by different stimuli [23]. Indeed, MSU crystal-induced NETs are distinct from PMA-induced NETs in terms of their components (e.g., actin-enriched structure) and resistance to nucleases. Thus, our present findings cannot be generalized to the clearance of NETs induced by different stimuli, and it will be interesting in the future to evaluate the detailed molecular mechanisms underlying the recognition and clearance of MSU crystal-induced NETs during gouty inflammation.

## Conclusion

Our results show that MSU crystal-induced NETs are engulfed by synovial fluid macrophages in patients with gouty arthritis, independent of the activation of the NLRP3 inflammasome. In synovial fluid, a significant negative correlation between the proportion of CD14^+^ cells and MPO-dsDNA complex was found. In addition, interaction with NETs was not associated with the induction of pro- and anti-inflammatory cytokines in CD14^+^ macrophages. Taken together, our data suggest that during a gout attack, synovial fluid macrophages may be involved in a self-resolving mechanism that clears NETs without inducing a significant immunological response.

## Supplementary Information

**Additional file 1: Supplementary Figure 1.** Flow cytometry gating strategy for the identification of CD14^+^ macrophages in the SFMCs of patients with gout. **Supplementary Figure 2.** MSU crystal-induced NET formation. Healthy neutrophils were incubated with MSU crystals (500 μg/ml) for 4 h and then checked for the expression of neutrophil elastase (red), myeloperoxidase (yellow), and citrullinated histone H3 (green) (a) or SYTOX Green (b). DNA was stained with Hoechst 33342 (blue). NET formation was visualized using immunofluorescent confocal microscopy. Magnification, 40×. **Supplementary Figure 3.** Engulfment ratio of NETs after culture with SFMCs in the presence of NLRP3 blockade. Engulfment ratio of NETs was determined as the relative fluorescence unit in SFMCs after incubation with or without SYTOX Green-labelled NETs. When indicated, cells were exposed to MSU crystals or CP-456773 (NLRP3 inflammasome inhibitor, 5 μM) (*n* = 10). * *P*-value < 0.05, ** P-value < 0.01. **Supplementary Figure 4.** Difference in the cytokine expression in synovial fluid CD14^+^ macrophages by serine protease inhibition during stimulation with or without NETs, MSU crystals, and/or LPS. When indicated, cells were exposed to PMSF (phenylmethylsulfonyl fluoride; serine protease inhibitor, 100 μM) (*n* = 6). * P-value < 0.05, ** P-value < 0.01, **** P-value < 0.0001.

## Data Availability

The data underlying this article are available in the article and in its online supplementary material.

## Abbreviations

MSU: Monosodium urate
NETs: Neutrophil extracellular traps
NE: Neutrophil elastase
MPO: Myeloperoxidase
TGF-β1: Transforming growth factor-β1
OA: Osteoarthritis
LPS: Lipopolysaccharide
BSA: Bovine serum albumin
SFMCs: Synovial fluid mononuclear cells
