# Supplementary material for: Neutrophil extracellular trap clearance by synovial macrophages in gout
Source: Arthritis Res Ther. 2021 Mar 19;23:88. doi: 10.1186/s13075-021-02472-4 (PMC7977263; doi:10.1186/s13075-021-02472-4)
Supplement: Supplementary file 1 — Additional file 1: Supplementary Figure 1. Flow cytometry gating strategy for the identification of CD14+ macrophages in the SFMCs of patients with gout. Supplementary Figure 2. MSU crystal-induced NET formation. Healthy neutrophils were incubated with MSU crystals (500 μg/ml) for 4 h and then checked for the expression of neutrophil elastase (red), myeloperoxidase (yellow), and citrullinated histone H3 (green) (a) or SYTOX Green (b). DNA was stained with Hoechst 33342 (blue). NET formation was visualized using immunofluorescent confocal microscopy. Magnification, 40×. Supplementary Figure 3. Engulfment ratio of NETs after culture with SFMCs in the presence of NLRP3 blockade. Engulfment ratio of NETs was determined as the relative fluorescence unit in SFMCs after incubation with or without SYTOX Green-labelled NETs. When indicated, cells were exposed to MSU crystals or CP-456773 (NLRP3 inflammasome inhibitor, 5 μM) (n = 10). * P-value < 0.05, ** P-value < 0.01. Supplementary Figure 4. Difference in the cytokine expression in synovial fluid CD14+ macrophages by serine protease inhibition during stimulation with or without NETs, MSU crystals, and/or LPS. When indicated, cells were exposed to PMSF (phenylmethylsulfonyl fluoride; serine protease inhibitor, 100 μM) (n = 6). * P-value < 0.05, ** P-value < 0.01, **** P-value < 0.0001. [file 13075_2021_2472_MOESM1_ESM.pptx]

## Slide 1
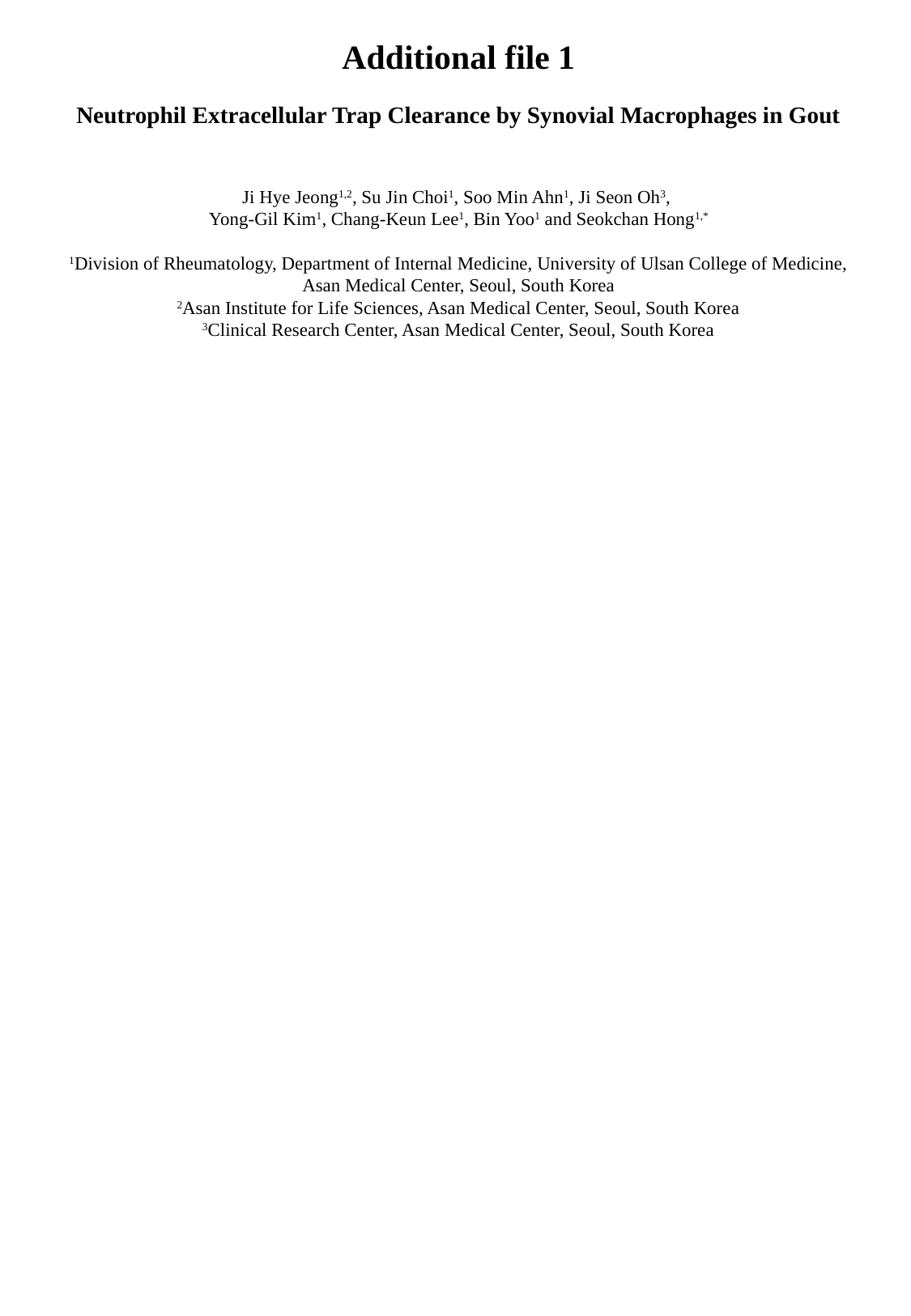

Additional file 1
Neutrophil Extracellular Trap Clearance by Synovial Macrophages in Gout
Ji Hye Jeong1,2, Su Jin Choi1, Soo Min Ahn1, Ji Seon Oh3,
Yong-Gil Kim1, Chang-Keun Lee1, Bin Yoo1 and Seokchan Hong1,*
1Division of Rheumatology, Department of Internal Medicine, University of Ulsan College of Medicine, Asan Medical Center, Seoul, South Korea
2Asan Institute for Life Sciences, Asan Medical Center, Seoul, South Korea
3Clinical Research Center, Asan Medical Center, Seoul, South Korea

## Slide 2
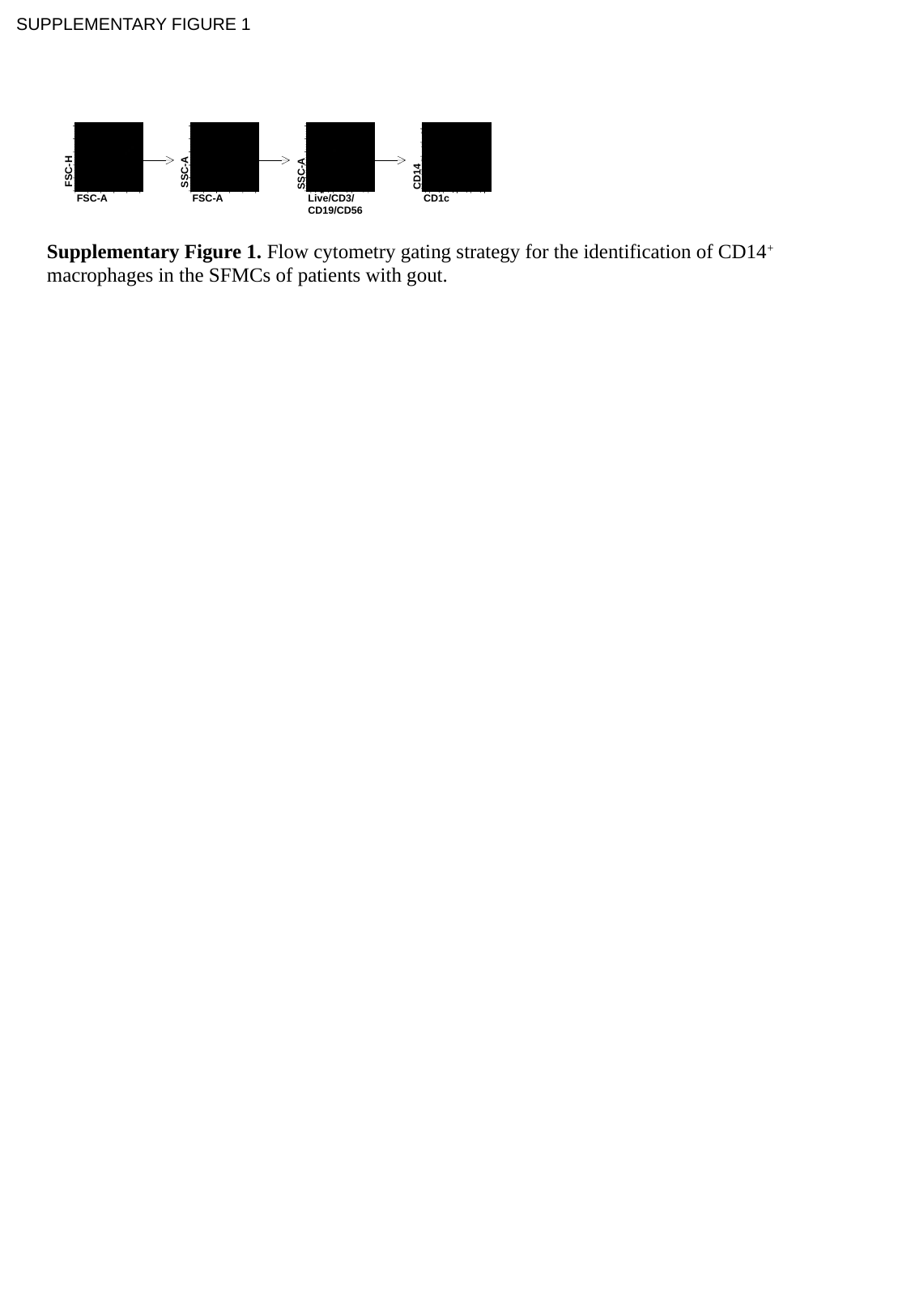

SUPPLEMENTARY FIGURE 1
FSC-H
SSC-A
CD14
SSC-A
FSC-A
FSC-A
CD1c
Live/CD3/CD19/CD56
Supplementary Figure 1. Flow cytometry gating strategy for the identification of CD14+ macrophages in the SFMCs of patients with gout.

## Slide 3
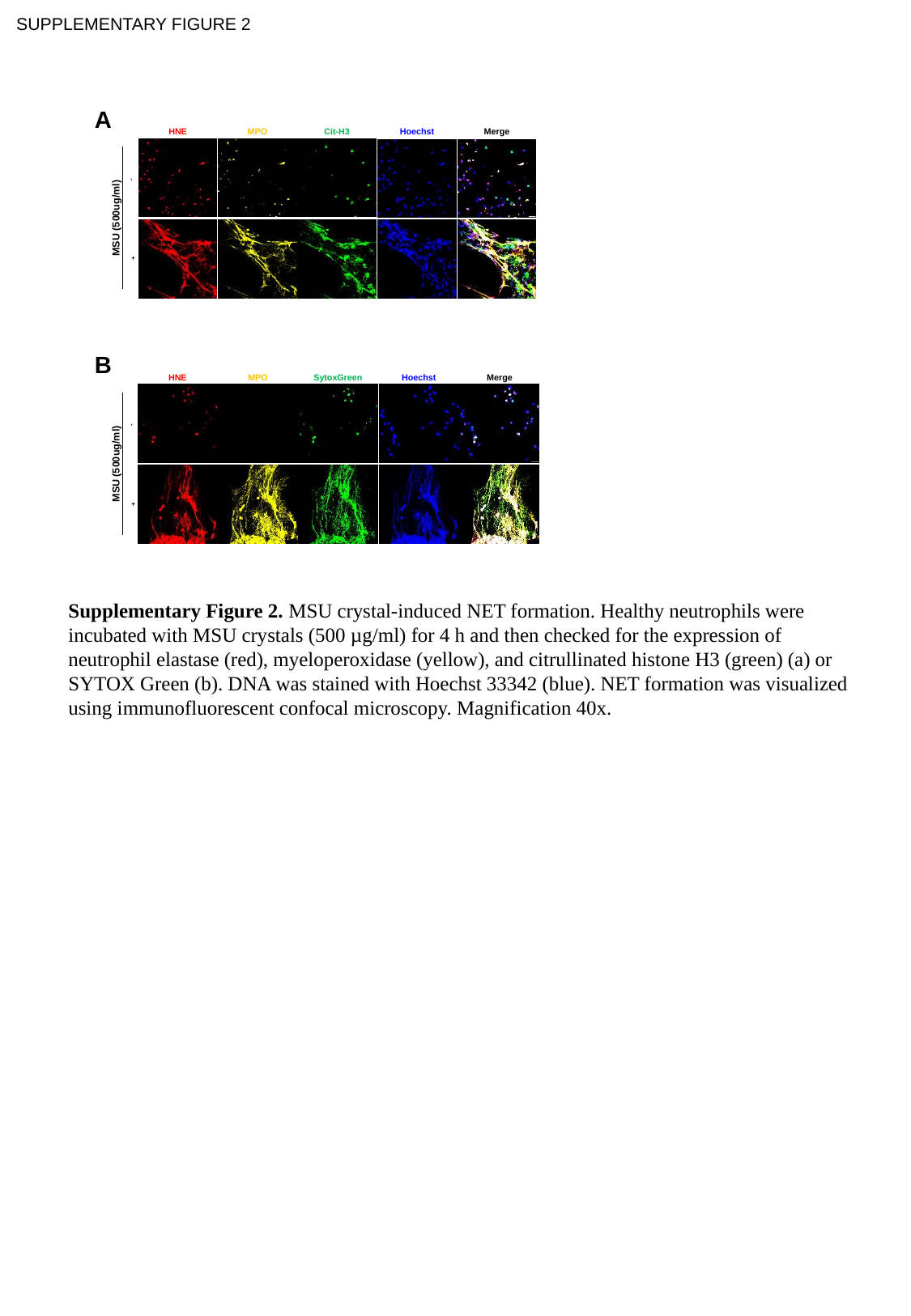

SUPPLEMENTARY FIGURE 2
A
HNE
MPO
Cit-H3
Hoechst
Merge
-
MSU (500ug/ml)
+
B
HNE
MPO
SytoxGreen
Hoechst
Merge
-
MSU (500ug/ml)
+
Supplementary Figure 2. MSU crystal-induced NET formation. Healthy neutrophils were incubated with MSU crystals (500 µg/ml) for 4 h and then checked for the expression of neutrophil elastase (red), myeloperoxidase (yellow), and citrullinated histone H3 (green) (a) or SYTOX Green (b). DNA was stained with Hoechst 33342 (blue). NET formation was visualized using immunofluorescent confocal microscopy. Magnification 40x.

## Slide 4
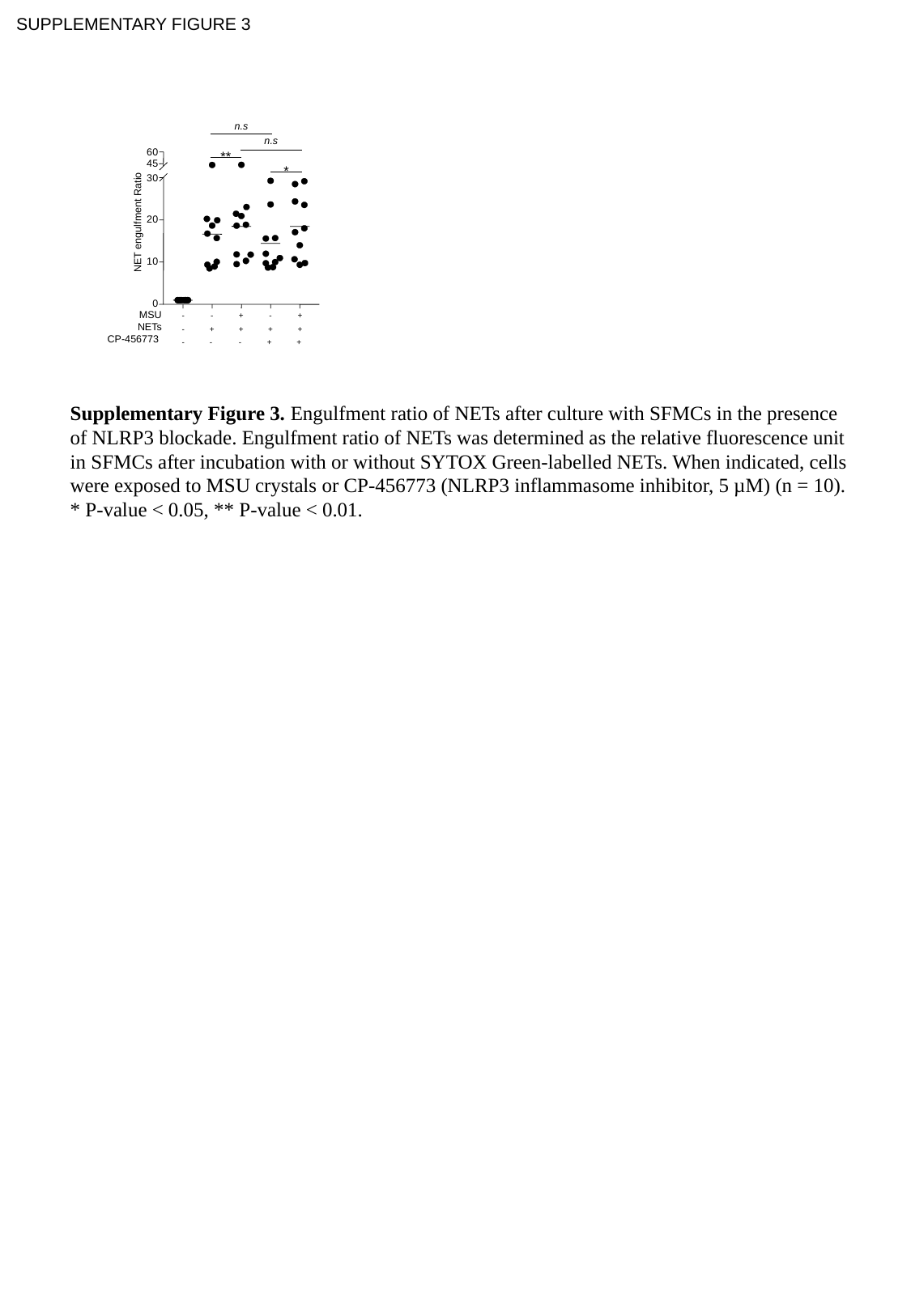

SUPPLEMENTARY FIGURE 3
n.s
n.s
60
45
30
20
 NET engulfment Ratio
10
0
-
-
-
-
+
-
+
+
-
-
+
+
+
+
+
MSU
NETs
CP-456773
**
*
Supplementary Figure 3. Engulfment ratio of NETs after culture with SFMCs in the presence of NLRP3 blockade. Engulfment ratio of NETs was determined as the relative fluorescence unit in SFMCs after incubation with or without SYTOX Green-labelled NETs. When indicated, cells were exposed to MSU crystals or CP-456773 (NLRP3 inflammasome inhibitor, 5 µM) (n = 10). * P-value < 0.05, ** P-value < 0.01.

## Slide 5
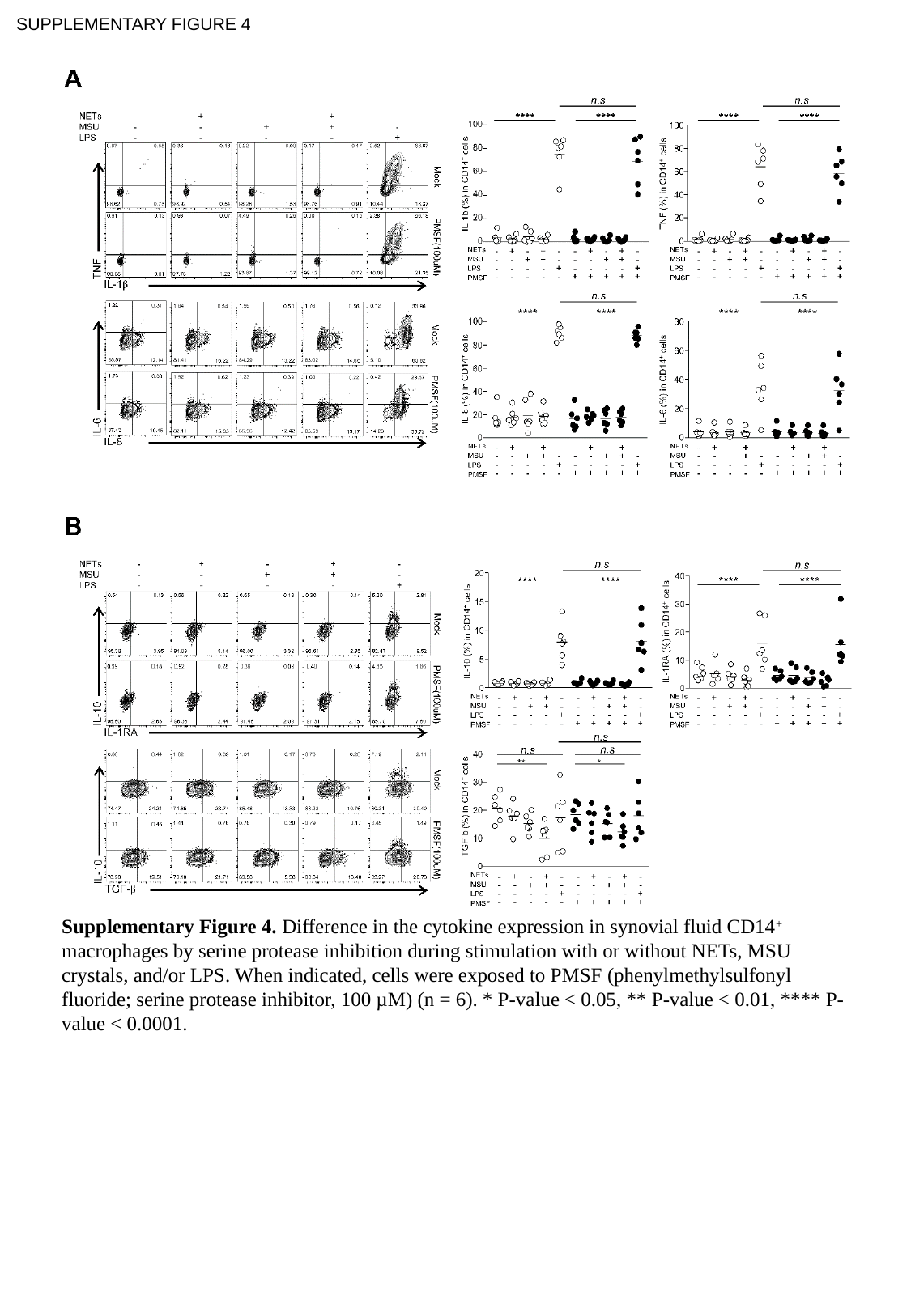

SUPPLEMENTARY FIGURE 4
Supplementary Figure 4. Difference in the cytokine expression in synovial fluid CD14+ macrophages by serine protease inhibition during stimulation with or without NETs, MSU crystals, and/or LPS. When indicated, cells were exposed to PMSF (phenylmethylsulfonyl fluoride; serine protease inhibitor, 100 µM) (n = 6). * P-value < 0.05, ** P-value < 0.01, **** P-value < 0.0001.
